# Investigation of peripheral inflammatory biomarkers in association with suicide risk in major depression

**DOI:** 10.1192/j.eurpsy.2024.1651

**Published:** 2024-08-27

**Authors:** B. Petho, T. Tenyi, R. Herold, P. Osvath, V. Voros, D. Simon, C. Molnar, M. A. Kovacs

**Affiliations:** ^1^Department of Psychiatry and Psychotherapy; ^2^Department of Immunology and Biotechnology, University of Pecs, Clinical Centre, Pecs, Hungary

## Abstract

**Introduction:**

Suicide is the most severe consequence of major depressive disorder (MDD). The most novel researches assume the role of immunological dysregulation in the background – several studies have reported alterations of inflammatory cells related to both MDD and suicidal behaviour (SB).

**Objectives:**

Changes in the number of certain immune cells and their ratios have been proposed as potential biomarkers of suicide risk (SR). The aim of our research was to investigate alterations of these values related not only to MDD as an assumed inflammatory state, but also to an increased risk of SB.

**Methods:**

In our restrospective cohort study carried out between January 2015 and January 2020, we investigated laboratory parameters of psychiatric patients diagnosed with MDD (*n*=101). Individuals with recent (≤48 hours prior) suicide attempt (SA) (*n*=22) and with past SA (>48 hours prior) (*n*=19) represented the high SR group. MDD patients with no history of SA (*n*=60) composed the intermediate SR group. We compared the number of neutrophil granulocytes, monocytes, lymphocytes, platelets, leukocytes, neutrophil-to-lymphocyte (NLR), monocyte-to-lymphocyte (MLR), platelet-to-lymphocyte ratio (PLR), red blood cell distribution width (RDW) and erythrocyte sedimentation rate (ESR). Furthermore, we evaluated alterations of these parameters related to antidepressant (AD) treatment, which has been proved to have anti-inflammatory effects. Statistical analyses were carried out using GraphPad 9.5.0 and MedCalc 16.8 programmes.

**Results:**

We found a significant increase in neutrophil granulocyte count (*p*=0.016), NLR (*p*=0.031, Fig. 1), monocyte count (*p*≤0.0001), MLR (*p*=0.005, Fig. 2), leukocyte count (*p*=0.048) and ESR (*p*=0.037) in patients with recent SA compared to patients with no history of SA. Moreover, there was a significant elevation in monocyte count (*p*≤0.0001), MLR (*p*=0.020, Fig. 3), ESR (*p*=0.041) and RDW (*p*=0.037) in patients with high SR compared to patients with intermediate SR. AD treatment resulted in a significant decrease in neutrophil granulocyte count (*p*=0.0163) and NLR (*p*=0.016), however, it did not affect the rest of the parameters.

**Image:**

**Figure:**
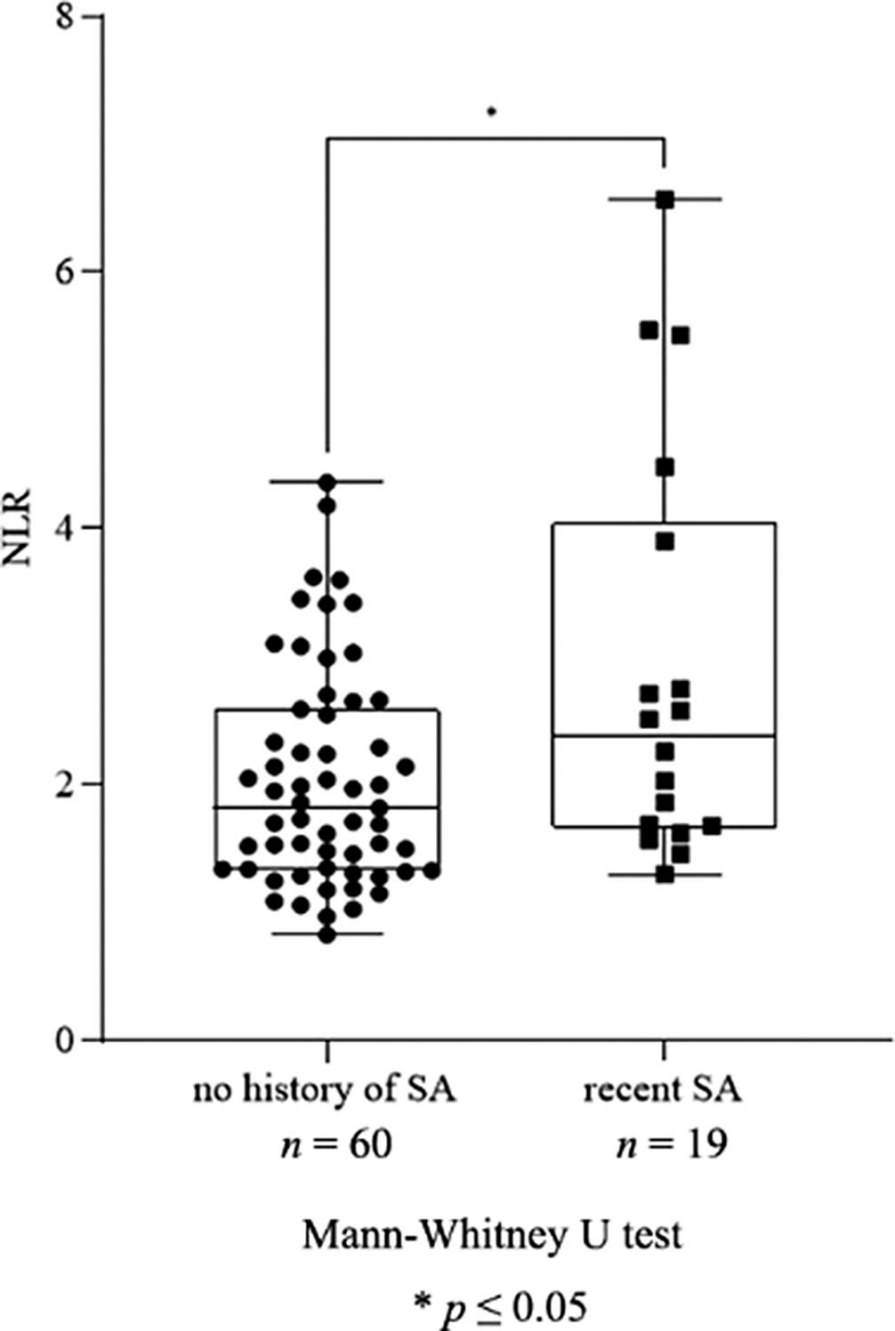

**Image 2:**

**Figure:**
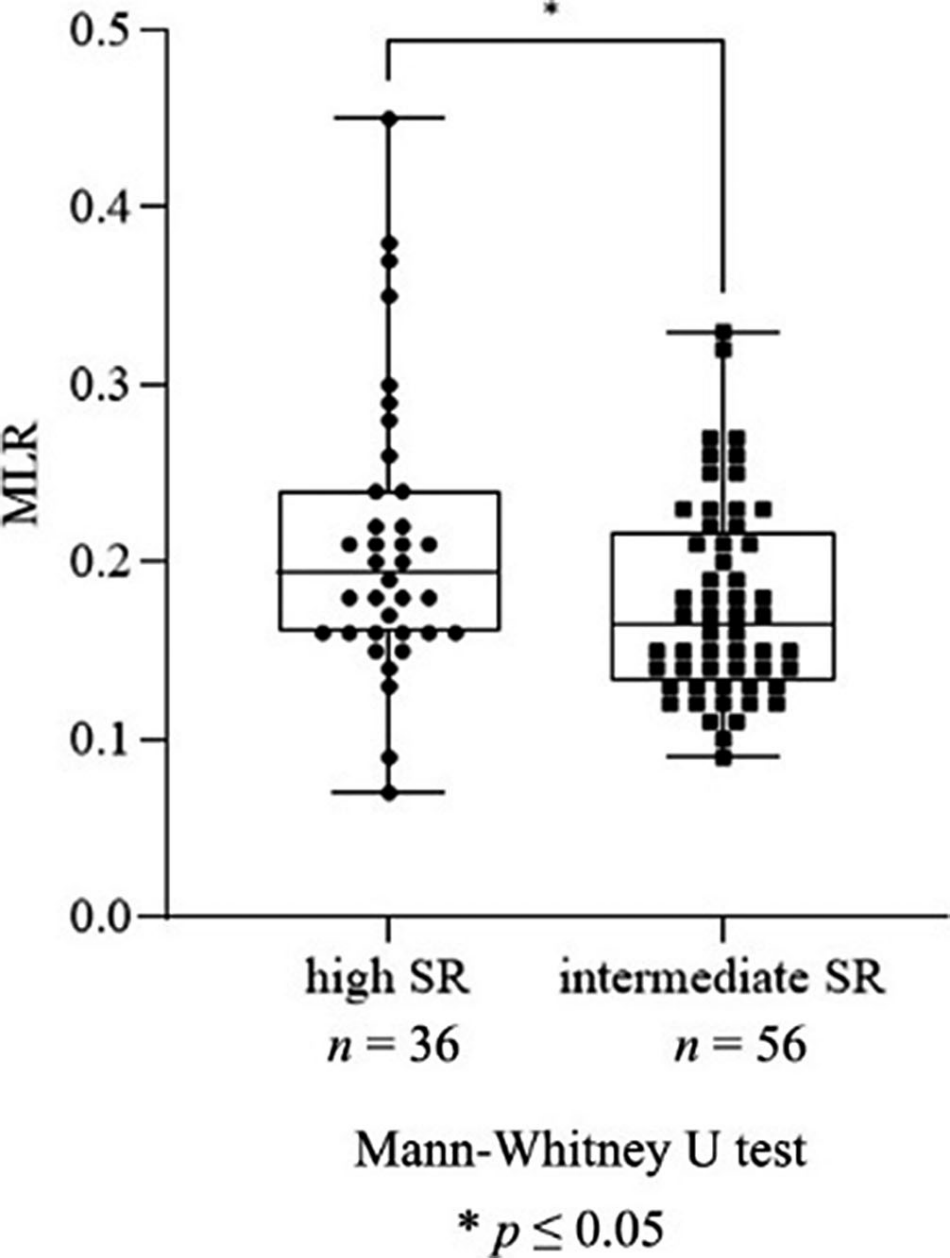

**Image 3:**

**Figure:**
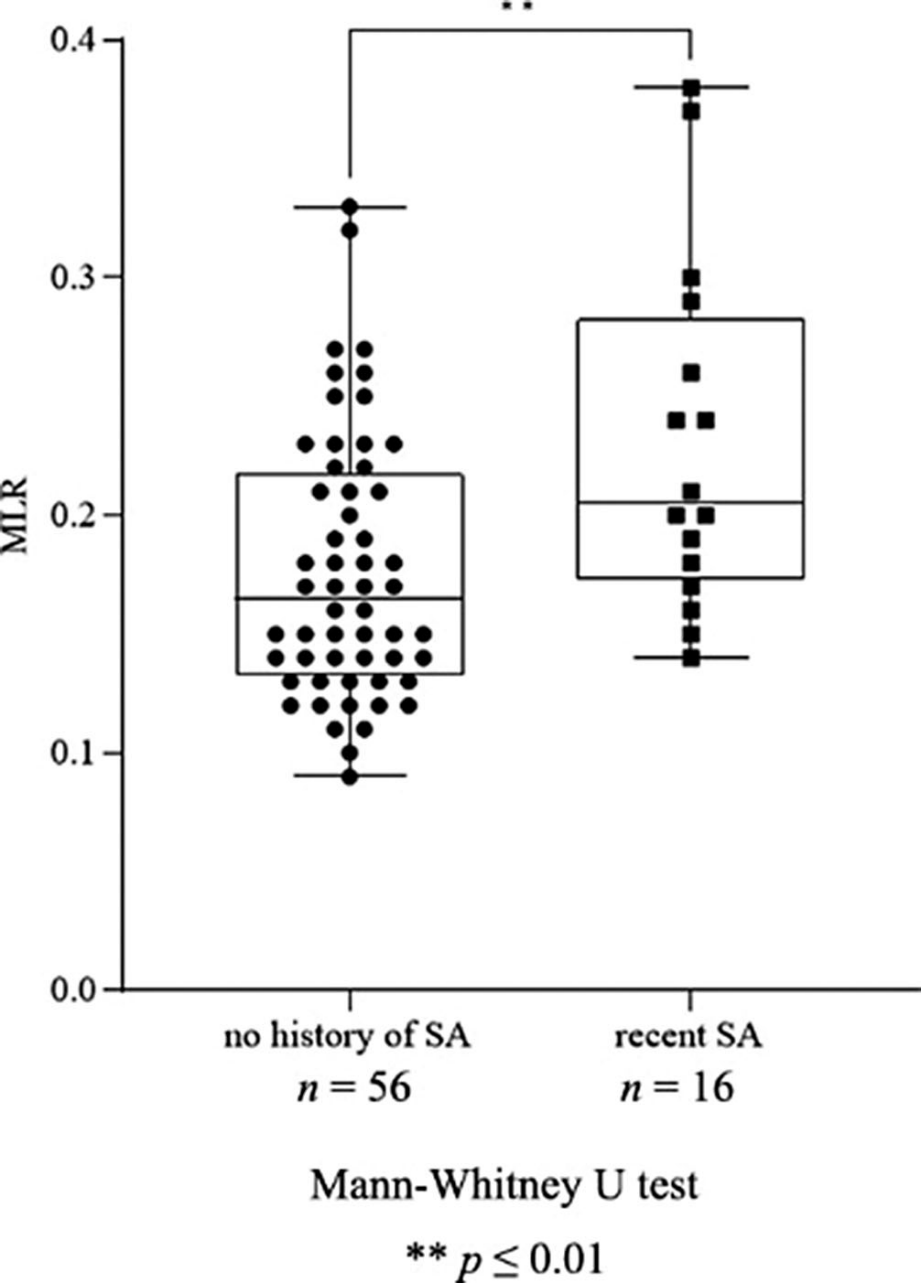

**Conclusions:**

Assuming immunological mechanisms in the background of MDD and SB, our findings support the role of NLR as a biomarker of acute SR, though its alterations may be masked by AD therapy in the long term. However, MLR – remaining unaffected by AD treatment – may be a possible indicator of both acute and long term suicidal vulnerability. In order to further specify the diagnostic value of these parameters, future prospective research is needed.

The study was supported by the FIKP-IV and the TNIL projects.

**Disclosure of Interest:**

None Declared

